# Adherence to the Mediterranean Diet Association with Serum Inflammatory Factors Stress Oxidative and Appetite in COVID-19 Patients

**DOI:** 10.3390/medicina59020227

**Published:** 2023-01-26

**Authors:** Mahsa Mohajeri, Reza Mohajery, Arrigo F. G. Cicero

**Affiliations:** 1 Digestive Disease Research Center, Ardabil University of Medical Sciences, Ardabil 56189-85991, Iran; 2 Energy Management Research Center, University of Mohaghegh Ardabili, Ardabil 56199-11367, Iran; 3 Medicine and Surgery Sciences Department, Alma Mater Studiorum University of Bologna, 40126 Bologna, Italy; 4 IRCCS AOU S. Orsola-Malpighi University Hospital, 40138 Bologna, Italy

**Keywords:** mediterranean diet, inflammatory factors, stress oxidative, appetite, COVID-19

## Abstract

*Background and Objectives:* The Mediterranean diet’s bioactive components are suggested to strengthen the immune system and to exert anti-inflammatory actions. This study investigated the association between adherence to the Mediterranean diet with serum inflammatory factors, total antioxidant capacity, appetite, and symptoms of COVID-19 patients. *Materials and Methods:* This cross-sectional study was conducted among 600 Iranian COVID-19 patients selected by a simple random method. The ten-item Mediterranean diet adherence questionnaire was used to assess diet adherence. At the beginning of the study, 5 cc of blood was taken from all patients for measurement of serum interleukin 1β) IL-1β), tumor necrosis factor (TNF-α), malondialdehyde (MDA), high sensitivity C-reactive protein (hs-CRP) and total antioxidant capacity (TAC). A human ELISA kit with serial number 950.090.096 produced by the Diaclone Company was used to test this cytokine using the sandwich ELISA method. *Results:* One hundred and five patients presented a high adherence and 495 patients presented a low adherence to the Mediterranean diet. The incidence of fever, cough, diarrhea, taste changes, and pneumonia severity index were significantly lower in patients who adhered to the Mediterranean diet more than other patients. Serum levels of tumor necrosis factor (5.7 ± 2.1 vs. 6.9 ± 2.8 *p* = 0.02), interleukin 1 beta (3.2 ± 0.02 vs. 4.9 ± 0.01 *p* = 0.02), high-sensitivity C-reactive protein (17.08 ± 4.2 vs. 19.8 ± 2.5 *p* = 0.03), and malondialdehyde (5.7 ± 0.2 vs. 6.2 ± 0.3 *p* = 0.02) were significantly lower in patients who adhered more to the Mediterranean diet than other patients. *Conclusion:* The Mediterranean diet can improve the symptoms and elevated serum inflammatory factors in COVID-19 patients, so clinical trial studies are suggested to confirm this effect.

## 1. Introduction

COVID-19 was identified as a pandemic by the World Health Organization on 11 March 2020 [1]. Optimizing] respiratory functioning is the major strategy, particularly in cases where the lower respiratory tract is involved [2,3]. The development of COVID-19 may be greatly influenced by inflammatory responses, according to the last studies’ results [4,5]. Rapid SARS-CoV-2 viral multiplication, cellular damage, and inflammatory responses can attract macrophages and monocytes and cause the production of cytokines and chemokines [6,7,8]. Cytokine storms and aggravations are induced following the attraction of immune cells and activation of immunological responses by these cytokines and chemokines. Several inflammatory indicators can be used to track and identify illness severity and mortality with some degree of accuracy. The high risks of developing severe COVID-19 are strongly associated with inflammatory markers such as procalcitonin (PCT), serum ferritin, erythrocyte sedimentation rate (ESR), C-reactive protein (CRP), and interleukin-6 (IL-6) [9,10]. Furthermore, it has been demonstrated that elevated serum amyloid A (SAA) levels play a role in the pathogenesis of COVID-19 and may be used as a biomarker to track the course of the illness. However, these findings are still debatable because other studies have not shown a change in the levels of IL-6, SAA, ESR, or CRP [11,12,13,14]. 

The Mediterranean diet (MD) is distinguished by a high intake of fruits, nuts, vegetables, legumes, and cereals (which in the past were largely unrefined), a high intake of olive oil but a low intake of saturated lipids, a moderately high intake of fish (depending on the proximity of the sea), a low-to-moderate intake of dairy products (and then mostly in the form of cheese or yogurt), a low intake of meat and poultry [15,16]. It is because of the preventive impact that the MD exhibits against a variety of chronic illnesses, including a beneficial effect on overall mortality, cardiovascular disease, and certain cancers, that high adherence to it has been associated with a higher health status [17,18]. The MD has also been suggested as one of the factors influencing these populations’ lifespans. The metabolic syndrome (MetS), certain of its components, and type 2 diabetes have been demonstrated to be negatively correlated with following a healthy eating pattern such as MD [19,20,21]. Due to the nutritious nature of this food pattern, it can also be effective in strengthening the immune system and preventing and controlling infectious diseases. An increasing body of research indicates that the MD’s anti-inflammatory qualities may contribute, at least in part, to its protective benefits [22,23,24].

Considering the importance of dietary pattern’s role in the prevention and control of COVID-19 complications, this study investigated the relationship between adherence to the Mediterranean diet and dietary inflammatory factors, appetite, and oxidative stress in COVID-19 patients.

## 2. Materials and Methods

### 2.1. Study Participants

**Figure 1:**
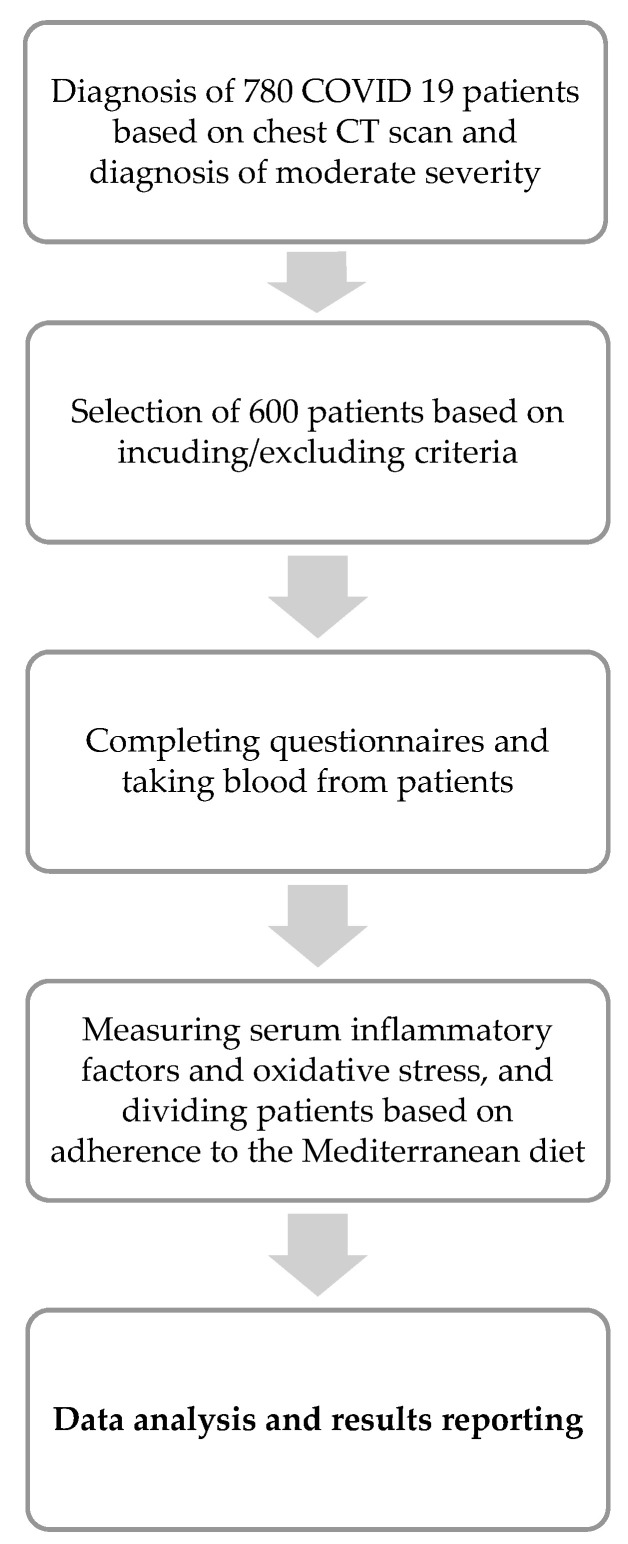
Study flow diagram.

This cross-sectional study was conducted among 600 COVID-19 patients aged ≥30 years old in Iranian hospitals. Sampling was carried out by a simple random method and using patient file numbers. Six hundred adult patients that met the inclusion criteria out of a total of 670 COVID-19 patients were enrolled in the research (Figure 1). The study included patients who were referred to the COVID-19 outpatient clinics of the Iranian hospital between January 2022 and March, having both calculated tomography (CT) scans of the thorax displaying moderate or severe involvement of the lower respiratory tract (as per radiologist diagnosis), and positive real-time reverse transcriptase–polymerase chain reaction (RT-PCR) tests in oro-nasopharyngeal swab samples. Patients with COVID-19 who had particular diseases, such as chronic liver or kidney diseases, or who were hesitant to participate in the study were excluded. Control patients with chronic diseases other than the skin-ones were also excluded from the study. 

The study was conducted according to the guidelines of the Declaration of Helsinki and approved by the Ethics Committee of the Ardabil University of Medicine Sciences on 17 January 2022 (IR.ARUMS.REC.1400.293). Informed consent was obtained from all subjects involved in the study. Written informed consent has been obtained from the patients to publish this paper.

### 2.2. Biochemical Measurements

At the beginning of the study, 5 cc of blood was taken from all patients for measurement of serum interleukin 1β) IL-1β), tumor necrosis factor (TNF-α), malondialdehyde (MDA), high sensitivity C-reactive protein (hs-CRP) and total antioxidant capacity (TAC). A human ELISA kit with serial number 950.090.096 produced by the Diaclone Company, Besançon, Frane, was used to test this cytokine using the sandwich ELISA method.

RANDOX’s RANSOM kit was utilized by the suggested procedure to assess the total antioxidant capacity employing spectrophotometry on an Abbot auto-analyzer at 600 nm. A particular substrate is incubated with peroxidase and hydrogen peroxide to form a radical substrate cation, which results in a persistent green-blue color that can be detected at a wavelength of 600 nm. This is the foundation for the measurement. The TAC is expressed in mmol/L. Immunoturbidimetric employed the Pars test diagnostic kit to measure the hs-CRP protein. Following the kit’s instruction manual, 200 L of reagent 2 comprising mouse monoclonal and goat polyclonal antibodies against human CRP antibody was added after placing 20 L of serum and 200 L of reagent 1 at 37 °C for 5 min. With the Abbot auto-analyzer set to 500 nm in mg/L, absorption was recorded at 30 and 90 s. Using kits from Bender Medical Systems, interleukins 1 were quantified by ELISA (Vienna, Austria). 

### 2.3. Appetite Assessment

A subjective assessment and an objective indicator for measuring appetite were taken into consideration in this study. To measure a subject’s sensations of hunger, desire to eat, and the likelihood of intake and fullness, a visual analog scale (VAS) was utilized (for subjective parameters). Food greasiness was added to the VAS to gauge the degree of food greasiness. To measure the experience before or after eating, a scale of 0 to 10 was used; the greater the number, the stronger the sensation. The subjects were in charge of their own experience.

### 2.4. Mediterranean Dietary Pattern Assessment

To calculate adherence to the MD, the ten-item, ten-point MEDAS (Mediterranean Diet Adherence Screener) [25], which has been scientifically verified, was used. With the use of the MEDAS questionnaire, the level of adherence to the MD was scored. Participants in the research were split into two groups based on their MEDAS scores: those who adhered to the MD less frequently (0–9) and those who adhered more frequently (10+). The frequency and proportion of replies that were positive to consuming each food were summed. 

### 2.5. Statistical Analysis

Statistical analyses were performed with STATA Version 14.0 for windows. All continuous variables were reported as the mean ± standard deviation. Inflammatory markers were compared between groups of adherence to the MD. Differences between group means were tested using an independent T-test. The Association between inflammatory markers and adherence to the MD was also assessed using multivariate regression. In all analyses, a *p*-value, of less than 0.05 was considered statically significant. Due to the difference in the study groups sample sizes, to validate the results of the T-test, the means difference of the variables and the confidence interval of all the variables were checked.

## 3. Results

Table 1 shows the general characteristics of the participants. None of the enrolled patients was vaccinated before infection. The mean ± SD of study patients was 52.9 ± 6.8 years old. Fifty percent of patients were male and 49% were female.

In Table 2, the results of positive answers to the MEDAS questionnaire are shown. In terms of positive response to the regular consumption of olive oil (97 vs. 67 *p* = 0.04), fruits (105 vs. 89 *p* = 0.04), vegetables (103 vs. 94 *p* = 0.02), legumes (104 vs. 79 *p* = 0.01), and fish/sea foods (101 vs. 61 *p* = 0.02), there was a significant difference between people who adhere more to the Mediterranean diet and people with less adherence to the Mediterranean diet. 

The status of symptoms and complications of COVID-19 based on the level of adherence to the Mediterranean diet is shown in Table 3. 

The incidence of fever [50 (47.6%) vs. 482 (92.5) *p* = 0.02], cough [38 (36.1%) vs. 380 (76.7), *p* = 0.01], dyspnea [62 (59.04) vs. 392 (79.1) *p* = 0.02], diarrhea [44 (41.9) vs. 280 (56.5) *p* = 0.02], taste changes [23 (21.9) vs. 365 (73.7), *p* = 0.05], blood pressure and pneumonia severity index [70.4 ± 6.3 vs. 73.4 ± 2.4, *p* = 0.04] were significantly lower in patients who adhered to the Mediterranean diet more than in patients with less adherence.

The results related to the comparison of appetite in the study patients are shown in Table 4. The amount of desire to eat was higher in patients with more adherence to the Mediterranean diet than in patients with low adherence (60% vs. 23% *p* = 0.02). About 31% of patients with greater adherence to the Mediterranean diet rarely felt full after eating, while only about 8% of patients with low adherence to the Mediterranean diet tended to eat more food after the main meal. 

The status of serum inflammatory markers in COVID-19 patients according to adherence to the MD has been shown in Table 5. Serum levels of TNF-α (5.7 ± 2.1 vs. 6.9 ± 2.8 *p* = 0.02), interleukin 1 beta (3.2 ± 0.02 vs. 4.9 ± 0.01 *p* = 0.02), hs-CRP (17.08 ± 4.2 vs. 19.8 ± 2.5 *p* = 0.03), and MDA (5.7 ± 0.2 vs. 6.2 ± 0.3 *p* = 0.02) were significantly lower in patients who adhered more to the Mediterranean diet than other patients. The level of serum total antioxidant capacity in patients with greater adherence to the Mediterranean diet (0.8 ± 0.02) was significantly higher than in other patients (0.6 ± 0.04) (*p* = 0.04). 

Table 6 shows the association of adherence to the MD with inflammatory markers in COVID-19 patients. There was a significant negative association between adherence to the Mediterranean diet and serum inflammatory factors in COVID-19 patients. The serum TNF-α in patients with more adherence to the MD was 1.32 units less than in other patients (coeff. = −1.32, *p* = 0.02). The serum level of hs-CRP in patients who adhered more to the Mediterranean diet was 1.89 units less than in other patients (coeff. = −1.89, *p* = 0.01). More adherence to the Mediterranean diet leads to a decrease of 1.34 units in the serum level of MDA (coeff. = −1.34, *p* = 0.04) and 1.08 units (coeff. = −1.08, *p* = 0.04) in the serum level of interleukin-1 beta in COVID-19 patients. The total antioxidant capacity in patients with greater adherence to the Mediterranean diet was 2.04 units higher than in other patients (coeff. = 2.04, *p* = 0.03).

**Figure 2:**
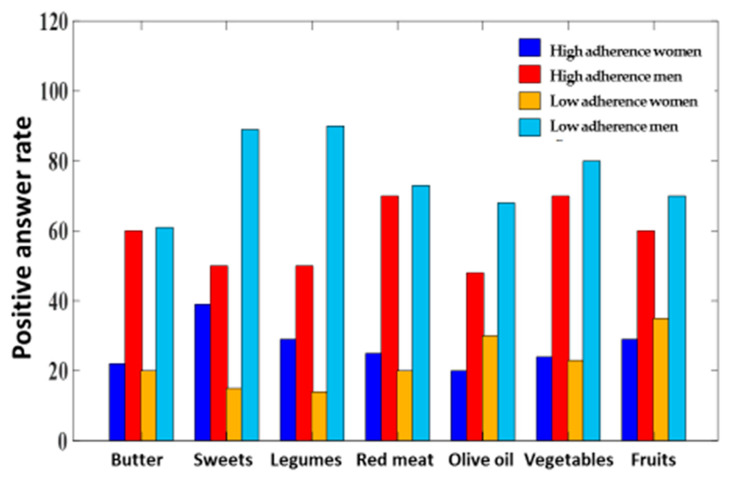
Self-reported dietary food class intake (Mediterranean diet questionnaire) in men and women involved in the study.

**Figure 3:**
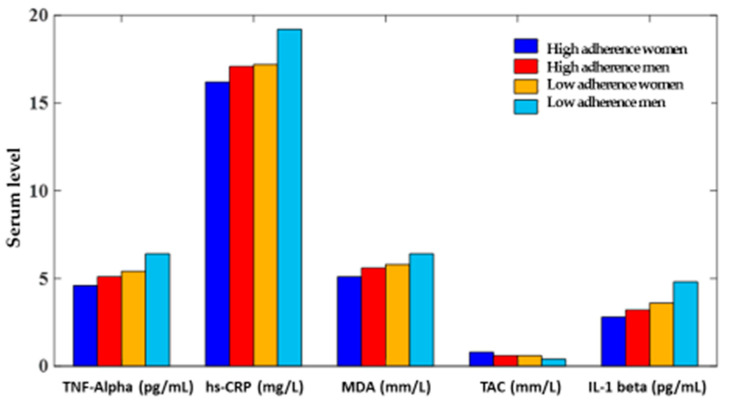
Serum level of inflammatory factors and total antioxidant capacity in study men and women.

Considering the intake of some specific food classes, the adherence of men to the Mediterranean diet is significantly lower than the one of interviewed women (*p* ≤ 0.05) (Figure 2). At the same time, the serum levels of the measured inflammatory parameters was significantly higher in men than in women (*p* ≤ 0.05), in particular in subjects non-adherent to the Mediterranean diet (Figure 3).

## 4. Discussion

For the first time, this study has shown that adherence to the Mediterranean diet has a significant negative relationship with the symptoms of COVID-19 and serum inflammatory markers among COVID-19 patients. Given the scientific plausibility supporting the positive benefits of an appropriate food intake on the immune system, it is hypothesized that a high-quality dietary pattern may offer protection against COVID-19. However, there is still little information about the association between long-term, sustained good food habits, such as the Mediterranean diet, and the risk of SARS-CoV-2 infection. Barrea et al. [26] pointed out the importance of the Mediterranean diet in improving the health of patients with COVID-19. He pointed out that the presence of olives, olive oil, fruits, and vegetables in this food pattern is one of the important things that affect the recovery process of COVID-19 patients. In another study, Greene et al. [27] examined the association between adherence to a Mediterranean diet and COVID-19 cases and deaths using an ecological study design. They observed that Mediterranean diet adherence was negatively associated with both COVID-19 cases and related deaths across 17 regions in Spain and that the relationship remained when adjusted for factors of well-being. They also observed a negative association between Mediterranean diet adherence and COVID-19-related deaths across 23 countries when adjusted for factors of well-being and physical inactivity. The anti-inflammatory properties of the Mediterranean diet are likely due to the polyphenol content of this diet. The study mentioned that there are confounding factors unrelated to dietary factors driving COVID-19 cases and related deaths. Perez-Araluce et al. [28] indicated that people with intermediate adherence to the Mediterranean diet had less risk of developing COVID-19. Another review study conducted by Anna Lucia Fedullo [29] showed that following the Mediterranean diet before and throughout pregnancy may have a protective impact by lowering gestational diabetes mellitus and gestational weight gain and enhancing the immune system’s response to viral infections such as as COVID-19. Inverse relationships have been shown between respiratory disorders, inflammation, and thrombosis with the Mediterranean diet, which includes olive oil, fish, honey, fruits, vegetables, and herbs. It is probable that a phytochemical mixture, such as those found in the Mediterranean diet, has stronger effects than a single molecule. It was indicated that chronic disease patients who follow a Mediterranean diet, as a whole, experience less PAF-induced platelet aggregation. The Mediterranean diet has been mentioned as a possible COVID-19 preventive diet, and it is stated that following this dietary pattern reduces mortality and duration of stay in hospital in patients ≥60 years old [30,31,32,33].

The increased serum levels of inflammatory factors are one of the important reasons for the COVID-19 incidence. Del Valle et al. [34] found that high serum interleukin-6, interleukin-8, and TNF-α levels at the time of hospitalization were strong and independent predictors of patient survival. While some inflammatory markers measures in this current study are acute phase reactants, some, such as total antioxidant capacity, may represent a chronic baseline state than acute change with a new illness. Yaghoubi et al. [35] reported that total antioxidant capacity levels were considerably lower in COVID-19 patients compared with healthy individuals (*p*  <  0.05) and also between patients with mild and severe diseases (*p*  <  0.05). Their findings suggest that COVID-19 patients may be susceptible to depleted total antioxidant capacity. 

The results of the present study confirmed the negative association between adherence to the Mediterranean diet and serum levels of hs-CRP, TNF-α, and interleukin-1β. Similar to our result, Sureda et al. reported that the high plasmatic inflammatory markers are closely correlated with low adherence to the Mediterranean dietary pattern [36]. Christina Chrysohoou [37] investigated how the Mediterranean diet affected blood levels of C-reactive protein, white blood cell counts, interleukin-6, tumor necrosis factor-α, amyloid A, fibrinogen, and homocysteine. His study results indicated that the levels of coagulation and inflammatory indicators were found to be lower in people who followed a conventional Mediterranean diet. The focus of a typical Mediterranean diet is on fresh, in-season vegetables, fresh salads, tomatoes, eggplant, cucumber, cabbage, rocket, radishes, garlic, onion, spinach, and lettuce are some examples of these. The most significant sources of phenolic compounds (mostly flavonoids) in the Mediterranean diet are vegetables. Vegetables also include dietary fiber, potassium, vitamin A, vitamin C, vitamin K, copper, magnesium, vitamin E, vitamin B6, folate, iron, thiamine, niacin, and choline, etc. [38,39]. Fruits and vegetables, legumes, olives, olive oil, and nuts are all parts of the Mediterranean diet that help blood inflammation reduction [40,41]. A dietary pattern with more fruits and vegetables has an association with low serum inflammatory factor levels. Corinna Koebnick et al. [42] evaluated the relationships of diet, obesity, and adipokine in Mexican Americans, and indicated that in comparison to those who consumed more fruits and vegetables and less sugar-sweetened beverages, those who had a diet high in sugar-sweetened beverages had greater levels of adiposity, CRP, leptin, and MCP-1 but lower levels of SFRP-5. Dietary patterns with more sugar-sweetened beverages but with fewer fruits and vegetable consumption cause high Adipokine profiles that lead to pro-inflammatory status. Other components of the Mediterranean diet are olive oil and olives. Consumption of these foods is associated with a decrease in inflammatory indicators [43,44]. In general, the results of all studies indicate the anti-inflammatory effect of the Mediterranean diet, so following this dietary pattern is recommended for the prevention and control of infectious diseases, especially COVID-19 [45].

Among other results of this study, there was a significant difference in the symptoms of COVID-19, including fever, cough, diarrhea, pneumonia severity index, and appetite among patients with different adherence to the Mediterranean diet. The incidence of COVID-19 symptoms in patients who adhered more to the Mediterranean diet was lower than in other patients. Consistent with our results, Perez-Araluce et al. [28] assessed the Mediterranean diet association with the risk of COVID-19 in the “Seguimiento Universidad de Navarra” cohort participants. This study results indicated that participants with intermediate adherence to the Mediterranean diet (3 < MDS ≤ 6) had significantly lower odds of COVID-19 incidence (multivariable-adjusted OR = 0.50, 95% CI: 0.34–0.73), and those with the highest adherence (MDS > 6) had the lowest risk (multivariable-adjusted OR = 0.36, 95% CI: 0.16–0.84, p for trend < 0.001) as compared with subjects with MDS ≤ 3. Angelis et al. [46]. mentioned that adherence to the Mediterranean diet has a main impact on cardiovascular diseases and other cardio-metabolic disorders, like diabetes that predisposes to COVID-19 infection and related outcomes.

This diet is distinguished by a combination of highly complex carbohydrates in fiber (found in cereals, legumes, vegetables, and fruits), polyunsaturated fatty acids with antiatherogenic and anti-inflammatory properties (found in olive oil and nuts), and bioactive substances with antioxidative properties such as flavonoids, phytosterols, terpenes, and polyphenols [37]. A well-balanced intake of micronutrients, such as vitamins and minerals, which are rich in this diet, helps to prevent malnutrition and immune deficiencies [32]. Several chemicals must be consumed together for proper immune system function. Nutrient-rich meals, such as MD, can reduce the elevated serum inflammation factors caused by nutrient-poor and high-calorie diets. Additionally, adhering to MD is associated with the restoration of the microbiota aerobiosis as Bacteroidetes and certain favorable Clostridium groups [47]. Considering that the composition of the intestinal flora indicates health, following a healthy food pattern such as the Mediterranean diet can strengthen the body’s immunity by maintaining the proper composition of the intestinal flora. [48] In fact, several nutritional intervention trials based on MD have collected the most important health benefits that this diet creates, including decreases in serum lipid levels; protection against oxidative stress; decreases in inflammation; platelet aggregation; modulation of hormones and growth factors implicated in cancer pathogenesis; and modulation of microbial metabolism, promoting the proper functioning of the host metabolism as well [49]. More research is being carried out now to prevent diseases, including cancer, CVD, metabolic disease, and even viral disorders. Here, we will give an overview of how MD’s most important elements affect the immune system’s regulation and the gut flora. MD is primarily characterized by its abundance of fruits, as well as by the availability of aromatic plants and spices to season food (dried herbs like oregano, rosemary, and thyme, for example), as well as seeds (cumin, sesame, etc.), olives, and nuts, all of which are high in a variety of polyphenols. Three important phenolic chemicals that are part of the MD are important to mention: hydroxytyrosol (HT), which is found in EVOO, resveratrol (RSV), which is found in red grapes, and quercetin (QUE), which is found in tea [23]. Higher HT concentrations decrease the levels of oxidized LDL and triglycerides and have a small effect on the expression of genes associated with oxidative-stress [50]. In high-fat diet (HFD)-induced obese mouse models, HT is still being investigated as a nutraceutical. It is being used to observe how this particular EVOO component reverses inflammatory parameters (elevated TNF-, IL-1, and IL-6) and inhibits the activation of TLR-4 and NK-kB pathways, which are related to intestinal permeability in obesity. The phenolic components in EVOO, such as HT, also boost the development of Bifidobacteria, which contribute to the anti-inflammatory effects in the gut. In general, it can be concluded that adherence to the Mediterranean diet and consumption of anti-inflammatory foods strengthen the body’s immunity and can be effective and important in the prevention, control, and treatment of symptoms and complications of chronic and even infectious diseases, especially COVID-19 [51]. 

The use of dietary questionnaire data and the cross-sectional design of this study were this study’s limitations, even though it was the first study that examined the association between adherence to the Mediterranean diet and inflammatory factors, appetite, and symptoms of COVID-19. An adequately powered, long-term, randomized clinical trial should be carried out to confirm our preliminary observation. Moreover, some inflammatory markers measured in this study are acute phase reactants, so in a next study, other markers like total antioxidant capacity (TAC) may better represent chronic baseline state than acute change with a new illness.

## 5. Conclusions

There is an inverse relationship between adherence to the Mediterranean diet and the symptoms and complications of COVID-19, and patients who followed the Mediterranean diet more in the past had less fever, cough, diarrhea, and lung infection. There was a negative relationship between adherence to the Mediterranean diet and serum inflammatory factors in COVID-19 patients. Long-term clinical studies among patients suffering from various infectious diseases, especially pneumonia and COVID-19, are needed to prove this relationship.

## Figures and Tables

**Table 1:** Demographic characteristics of the study patients.

Variables	Mean ± SD	Men *N* = 305	Women *N* = 295	*p* *
Age (y)	52.9 ± 6.8	51.8 ± 3.4	51.6 ± 6.4	0.08
Body mass index (Kg/m^2^)	29.5 ± 2.3	30.2 ± 2.8	28.4 ± 3.9	0.02
Smoking (%)	15 (35.83%)	215 (72.88%)	0	0.01 ^&^

*: Based on independent T-test ^&^: Based on chi-square test.

**Table 2:** Positive answers to the MEDAS questionnaire.

	Low Adherence to MD (*N* = 495)		High Adherence to MD (*N* = 105)		*p*
Olive oil, the main dressing	68	(13.73%)	98	(93.33%)	0.01
Olive oil, 4 ts/day	67	(13.53%)	97	(92.38%)	0.04
Vegetables, 2 s/day	94	(18.98%)	103	(98.09%)	0.02 *
Fruits, 3 s/day	89	(17.97%)	105	(88.57%)	0.04 *
Red meat, <1 s/day	95	(19.19%)	93	(88.57%)	0.07
Butter, <1 s/day	82	(16.56%)	81	(77.14%)	0.07
Sweet beverage, <1 s/day	89	(17.97%)	104	(99.04%)	0.05 *
Legumes, 3 s/week	79	(15.59%)	104	(99.04%)	0.01 *
Fish and seafood, 3 s/week	61	(12.32%)	101	(96.19%)	0.02
Sweets, <3 s/week	101	(20.40%)	102	(97.14%)	0.06
Nuts, 3/week	88	(17.77%)	104	(99.04%)	0.01 *
White meat over red	102	(20.60%)	104	(99.04%)	0.06

Notes: Positive answers to the MEDAS questionnaire. Data are expressed as numbers and percentages in parenthesis [n (%)] for categorical variables. Vegetables daily serving: 1 medium portion = 200 g; fruit daily serving: 1 serving = 100–150 g portion; red meat/hamburgers/other meat daily serving: 1 medium portion = 100–150 g; butter, margarine, or cream daily serving: 1 medium portion = 12 g; sweet or sugar-sweetened carbonated beverages daily serving: 1 medium portion = 200 mL; legumes weekly serving: 1 portion = 150 g; fish daily serving: 1 medium portion = 100–150 g; seafood daily serving: 1 medium portion = 200 g; nuts weekly serving: 1 portion of dairy product = 30 g. MEDAS: Mediterranean diet adherence screener; MD: Mediterranean diet; s: serving; ts: tablespoon; *: *p* < 0.05.

**Table 3:** Symptoms of COVID-19 in study participants according to adherence to the MD.

Symptoms	High Adherence *N* = 105 (%)	Low Adherence *N* = 495 (%)	*p* *
Fever	50 (47.6%)	482 (92.5%)	0.02
Cough	38 (36.1%)	380 (76.7%)	0.01
Dyspnea	62 (59.04%)	392 (79.1%)	0.02
Fatigue	98 (93.3%)	421 (85.05%)	0.03
Taste/smell abnormalities	23 (21.9%)	365 (73.7%)	0.05
Diarrhea	44 (41.9%)	280 (56.5%)	0.02
Systolic BP (mmHg) Mean ± SD	102.2 ± 12.1	120.3 ± 10.5	0.04 #
Diastolic BP (mmHg) Mean ± SD	77.3 ± 12.1	81.2 ± 9.8	0.01 #
Heart rate (/min) Mean ± SD	88.6 ± 4.2	87.9 ± 2.3	0.09 #
Respiratory rate (/min) Mean ± SD	18.6 ± 2.9	18.5 ± 2.9	0.8 #
Pneumonia Severity Index Mean ± SD	70.4 ± 6.3	73.4 ± 2.4	0.04 #

*: Based on chi-square test #: based on independent T-test.

**Table 4:** Comparison of appetite according to adherence to the MD.

Variables	High Adherence *N* = 105 *N* (%)	Low Adherence *N* = 495 *N* (%)	p *
Desire to eat	63 (60%)	114 (23%)	0.02
Satiety time after eating			
After eating a few tablespoons to a third of a plate	35 (33%)	248 (50.10%)	
After eating half of all the food served	30 (25.57%)	206 (41.61%)	
Rarely satiated	40 (30.09%)	41 (8.28%)	0.01
Time of feeling hungry			0.03
Never	18 (3.63%)	283 (51.17%)	
Low and sometimes	49 (65.71%)	104 (21.01%)	
The whole day	38 (36.19%)	108 (21.81%)	
Patients’ opinions about the taste of food			0.01
Bad and very bad	14 (13.33%)	264 (53.33%)	
Moderate	28 (26.66%)	181 (36.59%)	
Good and very good	42 (63%)	50 (10.10%)	

*: Based on the chi-square test.

**Table 5:** Status of serum inflammatory markers in COVID-19 patients according to adherence to the MD.

Adherence to MD (%)	TNF-α pg/mL	Hs-CRP Mg/L	MDA µM/L	TAC Mm/L	Interleukin 1 Beta (pg/mL)
High adherence *N* = 105 (%)	5.7 ± 2.1	17.08 ± 4.2	5.7 ± 0.2	0.8 ± 0.02	3.2 ± 0.02
Low adherence *N* = 495 (%)	6.9 ± 2.8	19.8 ± 2.5	6.2 ± 0.3	0.6 ± 0.04	4.9 ± 0.01
*p* *	0.02	0.03	0.02	0.04	0.02

*: Based on independent T-test.

**Table 6:** The association of adherence to the MD with inflammatory markers in COVID-19 patients.

Dependent Variables	Coeff.	95% CI	*p* *
**TNF-α**	−1.32	−1.28, −1.38	0.02
**Hs-CRP**	−1.89	−1.76, −1.92	0.01
**MDA**	−1.34	−1.22, −1.48	0.04
**TAC**	2.04	1.98, 2.14	0.03
**Interleukin 1 beta**	−1.08	−1.02, −1.15	0.04

*: Based on multivariate regression, adjusted to age and gender.

## Data Availability

Data is unavailable due to privacy or ethical restrictions.
